# Detection of a cardiac mass by [18F]FDG-PET/CT: a rare case

**DOI:** 10.3332/ecancer.2009.152

**Published:** 2009-08-19

**Authors:** A Mallia, LL Travaini, G Trifiro, G Paganelli

**Affiliations:** 1 Division of Nuclear Medicine, Ospedale San Paolo, Milan, Italy; 2 Division of Nuclear Medicine, European Institute of Oncology, Milan, Italy

## Abstract

Intra-cardiac masses present an important problem in cardiology. The differential diagnoses includes tumours, which may be primary (benign or malignant) or metastatic, and infected mural thrombi.

Myxomas, sarcomas, breast, lung and renal cancer represent the commonest causes of primary benign, malignant and metastatic intra-cardiac masses, respectively.

Recent studies have shown that cardiac involvement in malignant lymphoma is common but under-investigated.

Diagnostic imaging techniques for detection of cardiac masses include echocardiography, CT and MRI, with echocardiography having the highest sensitivity. We propose that 18-F-PET/CT may play an important role in the detection and evaluation of intra-cardiac masses.

## Case report

A 60-year-old gentleman was referred for an [^18^F]FDG-PET/CT to evaluate the relapse of a diffuse non-Hodgkin large B cell lymphoma initially localized in the right testis, retroperitoneal lymph nodes and right lung. Previous treatment included a right-sided orchidectomy and a right-sided lobectomy, chemotherapy and immunotherapy.

**Figure 1:**
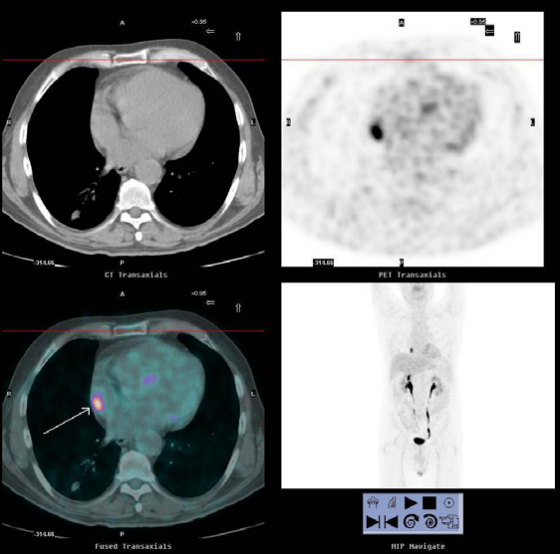
First [^18^F]FDG-PET/CT scan showing a suspicious uptake in the right atrium (SUV bw max 12).

**Figure 2:**
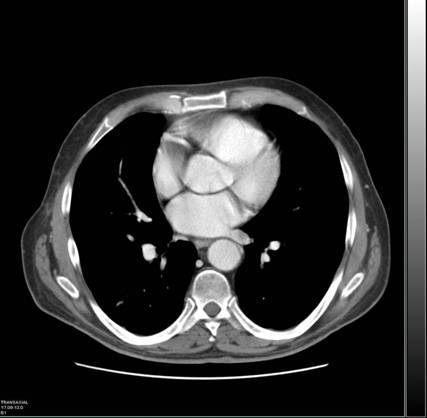
First CT scan performed in which no atrial lesion was reported.

An [^18^F]FDG-PET/CT scan showed no focal abnormalities apart from a dubious lesion in the right atrium (SUVbw max 12) (Figure1), which was not reported following a contrast enhanced CT scan (ceCT) performed one month earlier (Figure 2) and a second CT scan performed one month later.

**Figure 3:**
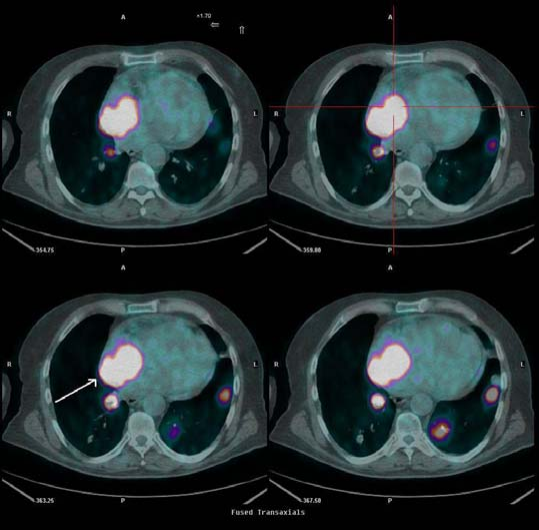
Second [^18^F]FDG-PET/CT scan, performed three months later, showing right atrial uptake (SUV bw max 24) together with pulmonary lesions.

**Figure 4:**
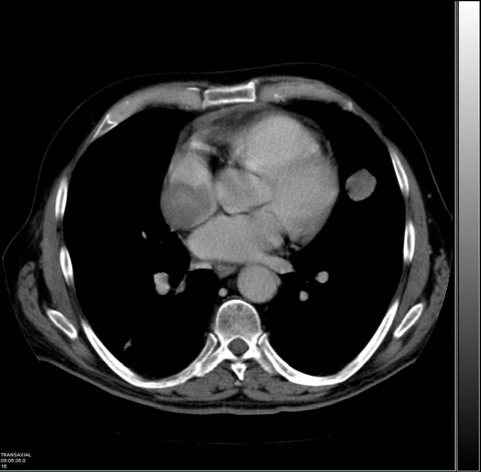
Second CT scan showing a right-atrial mass, measuring 5.7 cm in diameter together with pulmonary lesions.

[^18^F]FDG-PET/CT and ceCT scans were repeated approximately three months later. The [^18^F]FDG-PET/CT scan confirmed the presence of a right-atrial lesion which, when compared to the previous PET/CT scan, had increased in both size and uptake, with a maximal standardized uptake value (SUV bw max) now of 24. Pulmonary lesions were also reported (Figure 3). The ceCT scan showed a right-atrial mass of approximately 5.7 cm in diameter together with the pulmonary lesions (Figure 4).

A trans-thoracic echo Doppler further reported the presence of a dishomogenous hyperecogenic mass in the right atrium.

In view of the poor clinical condition of the patient, no histological diagnosis of the atrial mass was made although disease progression involving the heart was strongly suspected.

## Discussion

Intra-cardiac masses present an important problem in cardiology. The differential diagnosis includes tumours and infected mural thrombi.

Cardiac tumours are classified as primary benign or malignant tumours that arise from the heart, or as secondary metastatic tumours that invade the heart. Primary cardiac tumours occur with a low incidence. It is estimated that secondary metastatic tumours are a hundred times more common than primary cardiac lesions [1].

Over 75% of primary cardiac neoplasms are benign and are represented by myxomas and rhabdomyosarcomas. Myxomas are the most common, and 75% of these occurr in the left atrium, 20% are in the right atrium and the rest in the ventricles. Rhabdomyosarcomas are exceedingly rare and are more frequent in young patients. They typically involve the cardiac valves and invade the pericardium [2]. Other examples of benign cardiac tumours include haemangiomas, teratomas, lipomas, paragangliomas and pericardiac cysts.

Malignant primary tumours include sarcomas, pericardial mesothelioma and primary lymphomas. Sarcoma is the most common malignant and the second most common primary cardiac tumour affecting mainly middle-aged adults. Approximately, 40% are angiosarcomas [3]. Other types include undifferentiated sarcoma, malignant histiosarcoma, leomyosarcoma, fibrosarcoma, liposarcoma and osteosarcoma. Both pericardial mesothelioma and primary lymphoma are extremely rare with the latter usually occurring in immunocompromised patients.

Lung, breast and renal cancer together with soft-tissue sarcoma are the most common sources of metastasis to the heart [3]. Cardiac involvement in malignant lymphoma (as suspected in the case described above) is one of the least investigated areas of oncology [4]. A review of the current literature indicates that the incidence of cardiac involvement from lymphoma as identified by autopsy varies widely, ranging from 8.7% to 20% [5]. Although metastatic cardiac lymphoma can be symptomatic (arrhythmias, heart failure), clinical signs and symptoms are often absent or non-specific, and in most cases cardiac involvement in lymphomas remains undetected prior to the patient’s death.

Two-dimensional echocardiography is regarded as the primary diagnostic imaging technique for the evaluation of cardiac masses, with a diagnostic sensitivity of 93% for trans-thoracic echocardiography and 97% for trans-oesophageal echocardiography, although Qingyi *et al* reported a sensitivity of only 75.9% for the detection of cardiac metastasis [4].

Magnetic resonance (MR) has emerged as a useful tool for detailed evaluation of cardiac masses since it has a large field of view and allows for direct multi-planar imaging. An advantage of MR over echocardiography is its ability to obtain a tissue diagnosis in many cases [6].

Nuclear medicine techniques previously employed to detect cardiac tumours include blood pool imaging and gallium or thallium scintigraphy [7]. [^18^F]FDG-PET/CT has already been shown to be more accurate than CT in the evaluation of lymphomas and other tumours, especially in the follow-up of patients previously treated with chemotherapy and/or radiotherapy. [^18^F]FDG-PET/CT may have the advantage of detecting the tumour or metastases at an early stage, and in cases such as the one described above, it may help in the differentiation between benign and malignant lesions of the heart, which is difficult with other imaging modalities.

We report the first case of cardiac involvement in malignant lymphoma, which was diagnosed earlier by [^18^F]FDG-PET/CT than by ceCT. Early detection of metastatic cardiac involvement is important for proper diagnostic and therapeutic interventions.
